# Prompted Text-Based Vital Sign Recording Versus Unprompted Electronic Medical Record Entries in Patients With Advanced Heart Failure: Observational Study

**DOI:** 10.2196/71641

**Published:** 2026-09-09

**Authors:** Jenny Wu, Julia Chen, Lillian Min, Brandy Paul, Jenna M Keedy, Chandy Ellimoottil

**Affiliations:** 1 Department of Internal Medicine, University of Michigan, 1500 E Medical Center Dr, Ann Arbor, MI, United States, 1 734-936-4000; 2 Institute for Healthcare Policy and Innovation, University of Michigan, Ann Arbor, MI, United States; 3 Virtual Care Team, Michigan Medicine, Ann Arbor, MI, United States; 4 Quality Department, Michigan Medicine, Ann Arbor, MI, United States; 5 Department of Urology, University of Michigan, Ann Arbor, MI, United States

**Keywords:** remote patient monitoring, heart failure management, mobile text–based communication, patient engagement, telehealth

## Abstract

**Background:**

Long-term remote patient monitoring of weight, pulse, and blood pressure has been shown to significantly reduce mortality and hospitalization rates among patients with heart failure. Despite its proven effectiveness, maintaining patient engagement in remote monitoring programs remains challenging.

**Objective:**

This study aimed to evaluate the impact of 2-way text-based communication on prompting patients to record key vital signs and to compare it with unprompted patient reporting through electronic medical records in terms of engagement and clinical outcomes.

**Methods:**

We analyzed data from patients participating in the University of Michigan Advanced Heart Failure Program who reported daily weight, blood pressure, and pulse using either the MiChart Patient Outreach Texting Application (MPOTA) or patient enrolled flowsheets (PEFs). The study’s primary metric was the consistency of patient-reported vital signs, with secondary descriptive metrics including variations in hospitalization and emergency room visits pre-enrollment and postenrollment in the programs.

**Results:**

A total of 890 patients were included, with 301 enrolled in the MPOTA group and 589 in the PEF group. The engagement rate for the PEF group had a median of 2.29% (IQR 0%‐23.93%). In contrast, the MPOTA group showed a significantly higher median engagement rate of 66.67% (IQR 30.67%‐88.24%). There were no significant differences in hospitalization or emergency room visit rates across engagement categories (none, low, medium, and high) or between programs. Mean hospitalizations declined by 21% in the MPOTA group (from mean 0.53, SD 0.90 to mean 0.42, SD 0.88; *P*=.06) and 18% in the PEF group (from mean 0.50, SD 0.86 to mean 0.41, SD 0.80; *P*=.03) after the initiation of each program. This reduction was small and statistically significant only for the PEF group. Mean emergency room visits did not significantly change in either group. Regression analyses showed no significant association between engagement level and hospitalization or emergency room utilization, although medium engagement was associated with a nonsignificant trend toward fewer events. Despite improved engagement with MPOTA, this did not translate into significant reductions in hospitalizations or emergency room visits. All analyses of clinical outcomes were exploratory and underpowered, and no significant associations were found between engagement level and utilization.

**Conclusions:**

Although MPOTA was associated with substantially higher patient engagement levels compared to unprompted patient reporting, neither demonstrated significant differences in hospitalization or emergency room visits across engagement levels or between programs. Small reductions in hospitalizations were observed, but these were significant only in PEF, not in MPOTA. Observed changes in utilization were exploratory, small in magnitude, and underpowered to detect clinically meaningful effects. These findings suggest that mobile text-based communication may be a useful tool for improving engagement in remote monitoring programs for patients with advanced heart failure; however, further research is needed to assess its impact on clinical outcomes.

## Introduction

Heart failure affects an estimated 6.2 million individuals in the United States, with projections suggesting that the number of Americans with heart failure will exceed 8 million by the year 2030 [1-3]. Hospitalizations account for the majority of costs associated with heart failure, and these expenses are expected to increase [2]. Research shows that collecting weight, pulse, and blood pressure through long-term remote patient monitoring can be an effective way to reduce all-cause and cardiovascular mortality and hospitalizations in patients with heart failure [4]. Additionally, patients show interest in participating in self-monitoring of their condition [5]. Across heart failure remote patient monitoring studies, there are various ways to collect or prompt patient vitals, including video or telephone calls, 2-way mobile communications, and standalone devices that automatically transmit data [6,7]. Following the COVID-19 pandemic, social changes and advances in digital health technologies have increased interest in and the feasibility of remote monitoring strategies for chronic disease management [8].

However, the optimal method of reliably gathering patient information remains uncertain [9]. 2-way mobile communications (ie, texting) in heart failure have been feasible, well accepted, and may improve self-care, although their effects on clinical outcomes have been mixed [10]. This question is important because engagement is critical to the success of remote patient monitoring, yet the burden placed on patients differs between modalities. Lower-burden, easier-to-use communication modalities may reduce barriers associated with portal use, but their comparative impact on engagement in populations with heart failure remains unclear [11]. Understanding whether texting improves engagement over patient portal entry would advance current knowledge by isolating the impact of the communication methods themselves. Such insight can help health systems choose more effective and patient-centered strategies for remote patient monitoring program design.

In this study, we use the terms “2-way mobile communication” and “texting” interchangeably to refer to text messages that prompt patients to submit vital signs and enable direct responses through standard mobile text messaging. Moreover, mobile communication is attractive because most patients now have access to cell phones. Information on how patients can effectively submit information is critical because it can help guide future remote patient monitoring programs. Texting-based remote patient monitoring can improve compliance and engagement via reminders and participation in self-management [12]. Some studies show that texting-based programs can improve patient outcomes, reduce health disparities, and lead to higher uptake with greater satisfaction [13,14]. On the other hand, patients may find long-term adherence to such programs to be challenging [15], express concerns about associated costs [16], and encounter difficulties related to user fatigue [17].

Prior investigators examined the use of text-based data collection for patients with conditions outside of heart failure, such as mental health and diabetes [18,19]. These investigators found that texting-based data collection was helpful in addressing depression in adolescents and improving medication adherence in patients with diabetes [18,19]. However, these findings may not be generalizable to patients with heart failure because these prior studies either omitted vital sign monitoring or relied on weekly vital signs, which are insufficient for heart failure management [18,19]. Heart failure management depends on frequent, often daily, vital sign monitoring to detect early fluid retention or hemodynamic changes, and patients are often treated with medications such as diuretics or vasodilators that require timely titration based on physiologic trends [20]. These differences in monitoring intensity, medication responsiveness, and risk of rapid decompensation make it uncertain whether texting interventions that were effective in other conditions would be similarly effective for monitoring patients with heart failure.

Additionally, some studies combine mobile technology with a web-based application to process the information and respond to patients, such as systems that use automated Bluetooth data collection or mobile apps [21-23]. These combined methods may help reduce hospitalizations and emergency room visits, as well as improve self-care and satisfaction [21-23]. However, these studies either did not attempt to isolate the effect of separate components or did not focus on large populations with heart failure, and few have looked at remote patient monitoring for 6 months in patients with heart failure [7,9,24].

This observational study compares 2 concurrent remote patient monitoring programs for individuals with advanced heart failure. The key distinction between the programs is that one actively sends text messages to patients daily to prompt them to enter their vitals. In the other program, patients are instructed to enter daily vitals in a patient portal, but they are not prompted. Another difference is that the latter program requires patients to log in to their patient portal, whereas the first program allows patients to enter data directly into the electronic medical record through text messages. Existing literature supports the potential of mobile communication for improving self-management, but few studies have directly compared text-based prompting with unprompted patient reporting among patients with heart failure. This study aims to fill that gap by evaluating whether 2-way mobile communication improves engagement with remote monitoring. We also explore, in a descriptive manner, whether engagement level is associated with changes in hospitalization and emergency room utilization within our health system, recognizing that clinical outcomes cannot be comprehensively captured due to limitations of observational data and incomplete detection of events occurring outside the system.

## Methods

### Data Source and Study Population

Data for this study were collected from the electronic health records of patients enrolled in the University of Michigan Advanced Heart Failure Program. Patients in the Advanced Heart Failure Program have both heart failure with preserved ejection fraction and heart failure with reduced ejection fraction. Every patient enrolled in the Advanced Heart Failure Program is requested to report daily weights, blood pressure, and pulse through 1 of 2 mechanisms: the MiChart Patient Outreach Texting Application (MPOTA) program or patient enrolled flowsheets (PEFs). For this analysis, we used a study period from May 1, 2020, to August 20, 2022. We selected this study period to ensure that we had 6 months of data before and after the intervention start date. We excluded patients who were enrolled in either program but had missing (n=15) or erroneous data (n=1), or were enrolled in MPOTA but never received any text messages (n=15). Erroneous data refers to a single PEF patient whose calculated engagement rate exceeded 100%, indicating an invalid denominator and preventing reliable interpretation.

### Description of the Interventions

PEFs, launched in April 2015, have gone through multiple iterations and changes since their inception. They are an integrated feature of the electronic medical record accessible through the patient portal, allowing patients to manually enter their medical data by logging into their patient portal via a computer or mobile device. The PEF questionnaire consists of 10 categories, which include vitals, medications, general health, shortness of breath, swelling, gastrointestinal symptoms, events, diuretics, dietary sodium, and dietary fluid. The recommended frequency for patient responses to the PEF questionnaire is once a week, and patients are advised to report their vitals daily. Patients are responsible for remembering to answer these questions themselves or may receive reminders via phone calls. The Advanced Heart Failure Program staff felt that providing consistent reminders to patients via phone calls was labor-intensive. Therefore, in June 2019, the Advanced Heart Failure Program launched the MPOTA program as a tool to help facilitate data collection.

MPOTA simplifies data entry for patients by allowing them to fill in a subset of questions from the PEFs via text messaging. The platform is integrated with the institution’s electronic medical record, and all data collected are stored in the PEF. MPOTA consists of 6 questions inquiring about weight, systolic and diastolic blood pressure, pulse, missing any prescribed heart medications, and comparing symptoms to their last self-assessment. The care teams deem these 6 questions to be the most important part of the PEF and are also a subset of the 10 categories comprising the PEF questionnaire. Additionally, MPOTA sends a weekly reminder via text message to complete the flowsheet questions through the patient portal. To enroll patients in MPOTA, nurses must opt into the training program and enroll their patients. Nurses who choose not to use the MPOTA tool continue to use PEF with their patients. As a result, differences in nursing practices or resource availability may have influenced which patients participated in MPOTA versus PEF. In both groups, if a patient enters information that crosses a certain threshold, an in-basket notification is sent to a registered nurse.

### Primary and Secondary Outcomes

For this analysis, we compare the outcomes of patients enrolled in MPOTA to those who use the PEFs. The primary outcome is patient engagement, defined as the consistency of patient-entered vital signs after enrollment. MPOTA engagement rates are calculated by dividing the number of days a patient replies with information via text messaging by the number of days they receive a text. PEF engagement rates are calculated by dividing the number of days a patient enters information in the flowsheet by the number of days they are enrolled in the program. This approach was chosen because it reflects the available data for measuring engagement in each program, as a standardized time-based measure (eg, per week or per month) was not available for a direct comparison. However, we acknowledge that the use of different engagement definitions for MPOTA and PEFs may introduce bias and limit the validity of direct comparisons between the 2 groups. We also evaluate whether the level of patient engagement affects the number of hospitalizations and emergency room visits after enrollment in either program.

We evaluate 2 secondary outcomes: trends in hospitalizations and emergency room visits before and after enrollment in the 2 programs. To evaluate hospitalizations, we measure the change in the median number of hospitalizations in our health system 6 months before and after the program’s initiation. Our definition of hospitalizations includes both inpatient and observation stays. To assess the program’s impact on emergency room visits, we measure the median number of emergency room visits at our health system during the 6 months before and after the program’s implementation. We are unable to capture hospitalizations and emergency room visits that occur outside our health system. We use paired 2-tailed *t* tests to evaluate the difference in hospitalizations and emergency room visits before and after the intervention between the 2 groups.

### Statistical Analysis

Descriptive statistics were used to summarize demographic characteristics and study outcomes. Continuous variables were summarized using means or medians, as appropriate, and categorical variables were summarized using counts and percentages. Paired, 2-tailed *t* tests were used to compare the mean number of hospitalizations and emergency department (ED) visits during the 6 months before and after program enrollment within each program group. Two-way ANOVA was used to evaluate associations between program group, engagement category (none, low, medium, and high), and the number of hospitalizations or ED visits after enrollment. Logistic regression models were used to evaluate the association between program group and engagement category with the likelihood of any hospitalization or ED visit (yes or no) after enrollment. All statistical tests were 2-sided with statistical significance defined as *P*<.05. Descriptive statistics were obtained using Microsoft Excel. All inferential statistics were calculated using SAS (version 9.4).

### Ethical Considerations

This study was reviewed by the University of Michigan Institutional Review Board and determined to be exempt from ongoing review (HUM00228005). Informed consent was waived by the University of Michigan Institutional Review Board because the study met the criteria for exempt research involving secondary analysis of existing clinical data. All data were handled in compliance with institutional privacy and confidentiality standards.

## Results

### Description of Study Population

A total of 1515 patients were enrolled in MPOTA or PEF between February 2018 and February 2023. Patients enrolled before May 1, 2020, or after August 20, 2022, were excluded (n=588) to ensure that all patients had 6 months of data before and after the start of the program. Additional patients (n=37) were excluded for episodes other than heart failure (n=6), missing data (n=15), MPOTA patients not receiving a text (n=15), or other exclusions (n=1). After exclusions, there were a total of 890 patients included in the study, enrolled in either the MPOTA group (n=301) or the PEF group (n=589). The median days of enrollment in MPOTA was 372 (IQR 275‐609). The median days of enrollment in PEF was 395 (IQR 269‐508).

The mean age at the start of the programs was 62 (SD 15) years for the MPOTA group and 66 (SD 15) years for the PEF group (Table 1). There are 54.8% (165/301) male participants, 44.9% (135/301) female participants, and 0.3% (1/301) of unknown sex in the MPOTA group (Table 1). There are 57% (336/589) male participants and 43% (253/589) female participants in the PEF group (Table 1). The majority of patients are non-Hispanic Whites in both the MPOTA (221/301, 73%) and PEF (474/589, 80%) groups, with the MPOTA group having double the percentage of Black (or African American) participants (60/301, 20%) than the PEF group (60/589, 10%) (Table 1). Both the MPOTA and PEF groups have about one-third of patients with traditional Medicare, one-third with Medicare Advantage, and one-third with commercial insurance (Table 1).

**Table 1.** Demographics of MiChart Patient Outreach Texting Application (MPOTA) and patient enrolled flowsheets (PEFs) programs.

Characteristic		MPOTA (n=301)	PEF (n=589)
Sex, n (%)			
Male		165 (54.82)	336 (57.05)
Female		135 (44.85)	253 (42.95)
Unknown		1 (0.33)	N/A^a^
Race, n (%)			
White (non-Hispanic)		221 (73.4)	474 (80.5)
Black or African American		60 (19.9)	60 (10.2)
Asian or Pacific Islander		4 (1.3)	14 (2.4)
Hispanic		4 (1.3)	10 (1.7)
Other or unknown		10 (3.3)	22 (3.7)
Multiracial or multiethnic		2 (0.8)	9 (1.5)
Insurance, n (%)			
Medicare		88 (29.2)	196 (33.3)
Medicare Advantage		87 (28.9)	154 (26.1)
Medicaid		40 (13.3)	52 (8.8)
Commercial		83 (27.6)	182 (30.9)
Unknown		3 (1.0)	5 (0.9)
Age (y), mean (SD)		62 (15)	66 (15)
Days enrolled, median (IQR)		372 (275‐609)	395 (269‐508)

a Not applicable.

### Primary Outcome

The median engagement rate in PEF is 2.29% (IQR 0%‐23.93%) with a range of 0% to 99.76%. The median engagement rate in MPOTA is 66.67% (IQR 30.67%‐88.24%) with a range from 0% to 100%. The mean PEF engagement rate is 17.54% (SD 27.82%). The mean MPOTA engagement rate is 58.50% (SD 32.68%).

### Secondary Outcome

#### Hospitalizations

Approximately 66% of patients had zero hospitalizations before enrollment in either MPOTA or PEF (Table 2), while approximately 73% of patients had zero hospitalizations after enrollment in either program (Table 3). There was no change in the median number of hospitalizations 6 months prior to and 6 months post the program’s initiation for either the MPOTA or PEF groups. The median was zero hospitalizations (IQR 0‐1) for both groups pre-enrollment and postenrollment (Tables 2 and 3). The average number of hospitalizations for MPOTA declined by 21.4%, from 0.53 (SD 0.90) hospitalizations per patient before enrollment to 0.42 (SD 0.88) hospitalizations per patient after (*P*=.06, *df*=300). The average number of hospitalizations for PEF declined by a statistically significant 18.1%, from 0.50 (SD 0.86) hospitalizations before enrollment to 0.41 (SD 0.80) hospitalizations after (*P*=.03, *df*=588).

**Table 2.** Distribution of hospitalizations 6 months prior to program initiation.

Distribution, mean, and median of hospitalization before enrollment	Count of patients	
	MPOTA^a^ (n=301)	PEF^b^ (n=589)
Number of hospitalizations before enrollment, n (%)		
0	198 (65.8)	395 (67.1)
1	66 (21.9)	130 (22.1)
2	24 (8.0)	38 (6.5)
3	9 (3.0)	18 (3.1)
4	3 (1.0)	7 (1.2)
5	0 (0)	1 (0.20)
6	1 (0.30)	0 (0)
Hospitalization per patient^c^, mean (SD)	0.53 (0.90)	0.50 (0.86)
Hospitalization per patient, median (IQR)	0 (0‐1)	0 (0‐1)

a MPOTA: MiChart Patient Outreach Texting Application.

b PEF: patient enrolled flowsheet.

c *P* > |*t*| (*df*): .06 (300).

**Table 3.** Distribution of hospitalizations 6 months post program initiation.

Distribution, mean, and median of hospitalization after enrollment	Count of patients	
	MPOTA^a^ (n=301)	PEF^b^ (n=589)
Number of hospitalizations after enrollment, n (%)		
0	220 (73.1)	428 (72.7)
1	55 (18.3)	106 (18.0)
2	16 (5.3)	39 (6.6)
3	6 (2.0)	11 (1.9)
4	3 (1.0)	3 (0.5)
5	0 (0)	1 (0.2)
6	0 (0)	1 (0.2)
8	1 (0.3)	0 (0)
Hospitalization per patient^c^, mean (SD)	0.42 (0.88)	0.41 (0.80)
Hospitalization per patient, median (IQR)	0 (0‐1)	0 (0‐1)

a MPOTA: MiChart Patient Outreach Texting Application.

b PEF: patient enrolled flowsheet.

c *P* > |*t*| (*df*): .03 (588).

To examine the effect of different levels of engagement with the programs on the number of hospitalizations, engagement with the programs was classified into 4 groups: none (0%), low (0%‐33%), medium (33%‐66%), and high (66%‐100%). There was no significant difference in hospitalizations by program group, engagement category, or patient age (Table 4).

**Table 4.** ANOVA for the change in the number of hospitalizations.

Source	Type III SS^a^	Mean square^b^	*F* value (*df*)	*P*>*F*
Program group^c^	0.012	0.012	0.02 (1, 884)	.90
Engagement category^d^	1.036	0.345	0.51 (3, 884)	.68
Patient age	0.9636	0.963	1.41 (1, 884)	.24

a SS: sum of squares.

b The residual SD (√MSE) was 0.826.

c MPOTA (MiChart Patient Outreach Texting Application) or patient enrolled flowsheet (PEF).

d Percent engaged: none (0%), low (0%‐33%), medium (33%‐66%), and high (66%‐100%).

Due to a majority of patients enrolled in MPOTA and PEF had no hospitalizations before or after their enrollment (approximately 70%), differences in the mean number of hospitalizations can be difficult to detect. A regression analysis of any hospitalizations after enrollment (yes or no) can be useful to detect a difference. Neither program group (*P*=.74, *df*=1) nor engagement category (*P*=.54, *df*=3) showed significance in the model. The dose-response relationship also did not change consistently in one direction, and no individual engagement category was statistically significant. The effect increased when moving from no engagement to low engagement, and then decreased when moving to medium engagement. It returned to near zero for high engagement (Table 5). In this model, patient age was significant (*P*=.04, *df*=3). It is possible that sex or other demographic categories may also have an impact; however, they were not used in this regression.

**Table 5.** Regression analysis for any hospitalization.

Parameter	Estimate (SE)	Wald chi-square (*df*)	*P*>*χ*^2^
Intercept	1.677 (0.353)	22.51 (1)	<.001
Patient age	−0.011 (0.005)	4.129 (1)	.04
Program group^a^			
MPOTA^b^	0.032 (0.096)	0.109 (1)	.74
Engagement category^c^			
Low	0.029 (0.126)	0.053 (1)	.82
Medium	−0.204 (0.163)	1.558 (1)	.21
High	−0.017 (0.15)	0.013 (1)	.91

a MPOTA or patient enrolled flowsheet (PEF).

b MPOTA: MiChart Patient Outreach Texting Application.

c Percent engaged: none (0%), low (0%‐33%), medium (33%‐66%), and high (66%‐100%).

#### Emergency Room Visits

Approximately 91% of patients had zero emergency room visits before enrollment in either MPOTA or PEF (Table 6), and 91% of MPOTA and 93% of PEF patients had zero emergency room visits after enrollment in either program (Table 7). There was no change in the median number of emergency room visits 6 months prior to and 6 months post the program’s initiation for either the MPOTA or PEF group. The median was zero emergency room visits (IQR 0‐0) for both groups pre-enrollment and postenrollment (Tables 6 and 7). The average number of emergency room visits for MPOTA did not change, remaining at 0.12 (SD 0.43) emergency room visits per patient (*P*>.99, *df*=300). The average number of emergency room visits for PEF declined by 13.6%, from 0.11 (SD 0.46) emergency room visits per patient before to 0.10 (SD 0.45) emergency room visits per patient after (*P*=.44, *df*=588).

**Table 6.** Distribution of emergency room visits 6 months prior to program initiation.

Distribution, mean, and median of emergency room visits before enrollment	Count of patients	
	MPOTA^a^ (n=301)	PEF^b^ (n=589)
Number of ED^c^ visits before enrollment, n (%)		
0	273 (90.7)	538 (91.3)
1	22 (7.3)	43 (7.3)
2	3 (1.0)	5 (0.8)
3	3 (1.0)	2 (0.3)
7	0 (0)	1 (0.2)
Emergency room visits per patient^d^, mean (SD)	0.12 (0.43)	0.11 (0.46)
Emergency room visits per patient, median (IQR)	0 (0‐0)	0 (0‐0)

a MPOTA: MiChart Patient Outreach Texting Application.

b PEF: patient enrolled flowsheet.

c ED: emergency department.

d *P* > |*t*| (*df*): >.99 (300).

**Table 7.** Distribution of emergency room visits 6 months post program initiation.

Distribution, mean, and median of emergency room visits after enrollment	Count of patients	
	MPOTA^a^ (n=301)	PEF^b^ (n=589)
Number of ED^c^ visits after enrollment, n (%)		
0	274 (91.0)	548 (93.0)
1	20 (6.6)	32 (5.4)
2	5 (1.7)	6 (1.0)
3	1 (0.3)	2 (0.3)
4	1 (0.3)	0 (0)
7	0 (0)	1 (0.2)
Emergency room visits per patient^d^, mean (SD)	0.12 (0.45)	0.10 (0.45)
Emergency room visits per patient, median (IQR)	0 (0‐0)	0 (0‐0)

a MPOTA: MiChart Patient Outreach Texting Application.

b PEF: patient enrolled flowsheet.

c ED: emergency department.

d *P* > |*t*| (*df*): >.44 (588).

To examine the effect of different levels of engagement with the programs on the number of emergency room visits, engagement with the programs was classified into 4 groups: none (0%), low (0%‐33%), medium (33%‐66%), and high (66%‐100%). As with hospitalizations, there was no significant difference in emergency room visits by program group, engagement category, or patient age (Table 8).

**Table 8.** ANOVA for the change in the number of emergency room visits.

Source	Type III SS^a^	Mean square^b^	*F* value (*df*)	*P*>*F*
Program group^c^	0.121	0.121	0.6 (1, 884)	.44
Engagement category^d^	0.261	0.087	0.43 (3, 884)	.73
Patient age	0.091	0.091	0.45 (1, 884)	.50

a SS: sum of squares.

b The residual SD (√MSE) was 0.448.

c MPOTA (MiChart Patient Outreach Texting Application) or patient enrolled flowsheet (PEF).

d Percent engaged: none (0%), low (0%‐33%), medium (33%‐66%), and high (66%‐100%).

As with hospitalizations, over 90% of patients enrolled in MPOTA and PEF had no ED visits before or after their enrollment, and thus differences in the mean number of ED visits can be difficult to detect. Similar to hospitalizations, a regression analysis of any ED visits after enrollment (yes or no) can be useful for detecting a difference. Neither program group (*P*=.30, *χ*^2^_1_) nor engagement category (*P*=.64, *χ*^2^_3_) showed significance in the model. The dose-response relationship did not change consistently in one direction, and no individual engagement category was statistically significant. The effect increased from no engagement to low engagement, and then decreased when moving to medium engagement. It increased again for high engagement (Table 9). In this model, patient age was not significant (*P*=.88, *χ*^2^_1_). It is possible that sex or other demographic categories may also have an impact; however, they were not used in this regression.

**Table 9.** Regression analysis for any emergency room visits.

Parameter	Estimate (SE)	Wald chi-square (*df*)	*P* >*χ*^2^
Intercept	2.504 (0.567)	19.472 (1)	<.001
Patient age	−0.001 (0.009)	0.021 (1)	.88
Program group^a^			
MPOTA^b^	−0.162 (0.155)	1.095 (1)	.30
Engagement category^c^			
Low	0.028 (0.211)	0.017 (1)	.90
Medium	−0.273 (0.252)	1.18 (1)	.28
High	0.252 (0.257)	0.962 (1)	.33

a MPOTA or patient enrolled flowsheet (PEF).

b MPOTA: MiChart Patient Outreach Texting Application.

c Percent engaged: none (0%), low (0%‐33%), medium (33%‐66%), and high (66%‐100%).

## Discussion

### Principal Results

In terms of engagement rates, the MPOTA group demonstrates a considerably higher median engagement rate than that of the PEF group. However, this difference must be interpreted with caution, as the 2 programs use distinct engagement definitions that are not directly comparable. Similarly, the MPOTA group displays a notably higher mean engagement rate as well. However, this increased engagement did not correspond to a significant change in the median number of hospitalizations or emergency room visits before and after program initiation. These findings suggest that while text-based patient monitoring may enhance day-to-day engagement, it did not lead to measurable differences in short-term clinical outcomes in this study. Consistent with the descriptive nature of this analysis, these outcome comparisons were not designed to assess causal effects of either program.

The mechanism behind this enhanced engagement may be multifactorial. We hypothesize that the convenience of receiving automated text reminders, combined with the ease of responding via text messaging, contributed meaningfully to patient engagement. Unlike the approach used in the PEF cohort, which required patients to log into a patient portal, the system used in the MPOTA cohort was integrated into patients’ existing communication habits, thereby reducing barriers to engagement. This may be valuable for older adults or individuals less familiar with technology, as text messaging may offer a more accessible alternative to app-based or portal-based platforms. Although our study did not include a qualitative component, future research could explore patient and provider perspectives to better understand preferences and user experiences.

These findings should be interpreted in the context of our study population, which was largely non-Hispanic White and included a high proportion of patients with Medicare coverage. While these characteristics reflect the demographics of our health system, they may limit generalizability to the broader population with heart failure. The effectiveness and acceptability of text-based remote monitoring programs like MPOTA may vary in other settings. Future studies should investigate how similar interventions perform in more diverse populations to better understand their broader applicability.

### Limitations

First, our study only captures the documentation of hospitalizations and emergency room visits specifically within the confines of our health system. Our research focuses on alleviating congestion within the ED of our health system. Patients in either group may have sought care outside of our health system, such as at external hospitals or urgent care centers, which would not be reflected in our data. While we found no significant difference in hospitalization or emergency visit rates between the 2 groups, this potential underreporting remains a limitation in interpreting these results. However, we do not have reason to believe that care sought outside our health system would have disproportionately affected one group over the other. Future studies could consider linking data across health systems.

Second, the difference in definitions for patient engagement between the PEF and MPOTA cohorts makes them not directly comparable. Our approach was determined by data availability, as we did not have access to a standardized time-based measure (eg, engagement per week) for either group. Consequently, MPOTA patient engagement may be higher due to the responsiveness measure, derived from the ratio of days a patient responds to received texts. In contrast, patient engagement for PEF is determined by mean usage over time, calculated as the ratio of days a patient enters data over their total enrollment period. This difference in methodology between the 2 programs may have contributed to higher engagement rates in the MPOTA cohort compared to PEF, where engagement is based on total enrollment time. Notably, PEF patients receive instructions and encouragement to consistently record vital signs at the onset of the study. Accordingly, the higher engagement observed in MPOTA may partly reflect these definitional differences rather than true behavioral differences. Future studies should adopt a consistent time-based engagement metric to allow for more valid comparisons.

Third, enrollment in the MPOTA program was based on nurse opt-in and discretion, which may have introduced selection bias. Nurses may have selected patients they perceived as more likely to engage with or benefit from the program, potentially inflating observed engagement rates in this group. This selection could have been influenced by factors such as patient motivation, health literacy, or access to mobile technology—characteristics that were not measured in our analysis. Because enrollment criteria were not standardized, we were unable to adjust for these differences. Together, these factors introduce the possibility of confounding bias that we were unable to fully address within the constraints of the available data. Future work should consider randomizing or standardizing enrollment to reduce bias.

Fourth, although both cohorts consisted of patients with advanced heart failure managed in the same clinic, unmeasured confounding factors—such as social determinants of health, transportation access, frequency of provider interactions, or underlying disease severity—may have influenced outcomes like hospitalization and emergency room utilization. Patients in either program may have also been engaged in other care programs at our health system, such as transitions of care or chronic care management. These variables were not captured in our analysis and may help explain the absence of significant changes in clinical outcomes. Incorporating these variables in future studies may help clarify whether remote monitoring interventions have differential effects across subpopulations.

Fifth, while both cohorts were drawn from the same clinic, the overall sample predominantly included non-Hispanic White patients, which may limit the generalizability of our findings to more diverse or underserved populations. Notably, the MPOTA cohort included a significantly higher proportion of Black or African American patients compared to the PEF cohort. These demographic differences reflect the patient population served by our health system. Future work involving more diverse populations would help determine the broader applicability of these findings.

Despite these limitations, our findings suggest that text messaging may be a promising tool to increase patient engagement. Although we did not observe changes in hospitalizations and emergency room visits, the significantly higher engagement rates associated with text messaging may still hold clinical relevance. For instance, when timely data such as vital signs are needed for medication titration, text messaging may offer a more reliable method of data collection than patient-entered information through the patient portal.

### Comparison With Prior Work

The enhancement of engagement rates with MPOTA is congruent with other studies suggesting that lower-burden text messaging approaches may facilitate patient engagement [25]. In one study, patients recently discharged after acute decompensated heart failure were enrolled in a 30-day text message-based self-management program [7]. The study population was predominantly African American and low-income [7]. Text messaging in this study was associated with high rates of satisfaction and a significant improvement in management and maintenance among patients with acute decompensated heart failure [7]. Their findings may be partly influenced by contextual factors, including the enrollment of patients before discharge for acute decompensation, when patients may be more receptive to health interventions. This difference may account for the stronger improvements in self-management observed in that study. However, their small sample size and lack of a control group also limit the generalizability of their results.

Our finding that the text messaging platform does not change hospitalizations and emergency room visits contrasts with other studies. Similar to the prior example, another study found that readmissions were significantly lower in the 6 months after discharge in the text messaging and telephone intervention groups than in the usual care group for patients with chronic heart failure [26]. The time frame of 6 months is similar to our study period; however, in that study, patients were enrolled upon hospital admission, a period when patients may be at higher risk for readmission and more receptive to follow-up interventions [26]. In contrast, our patients were enrolled when they joined our outpatient heart failure program, which may correspond to a lower baseline hospitalization event rate. These contextual differences may help explain why prior interventions observed reductions in hospitalizations, whereas MPOTA, implemented in a lower-risk outpatient population, did not.

A systematic review synthesized multiple randomized and observational studies evaluating text messaging and mobile app interventions for the secondary prevention of cardiovascular disease, including coronary heart disease, cerebrovascular disease, and heart failure [27]. Across these populations, text messaging interventions were generally associated with high patient satisfaction [27]. The review found that successful interventions often featured greater engagement through 2-way messaging, higher message frequency, and personalization [27]. These factors may help explain the higher engagement rates observed in the MPOTA cohort, which used automated daily text prompts that integrated into patients’ existing communication habits [28]. Although our study did not show changes in short-term clinical outcomes, it aligns with broader evidence that text messaging can meaningfully support patient engagement and self-management in chronic disease management [29].

Building on this broader evidence, a more recent large randomized clinical trial evaluated a 30-day automated text messaging program for patients recently discharged from acute care hospitals across 30 primary care practices [30]. Similar to MPOTA, the intervention achieved high engagement (79.5% responded to at least 1 message) and satisfaction, but it did not significantly reduce 30-day ED visits or readmissions [30]. One key difference is that their study population included medium-risk to high-risk adults with a range of medical conditions, rather than exclusively patients with heart failure [30]. These findings suggest that high patient engagement with text messaging may be achievable across various patient populations. Although short-term clinical outcomes such as readmissions may remain unchanged, the scalability and low patient burden of automated text-based systems may still provide value by supporting routine monitoring, facilitating timely interventions, and integrating into patients’ communication routines.

Furthermore, a poster presentation reported that text messages yield greater patient engagement compared to notifications from a smartphone app, even when participants were trained to use the app [31]. The cumulative insights gleaned from our investigation, in conjunction with these studies, underscore the potential of text messaging to support engagement among patients with heart failure, which may be an important component of management.

### Conclusions

The findings from this study suggest that while the adoption of the MPOTA system did not result in a noticeable reduction in hospitalizations or emergency room visits, it significantly improved patient engagement levels. Because engagement was measured differently across programs, these differences may reflect relative patterns within each system rather than a standardized comparison. Even so, the results underscore the potential of mobile text-based communication to improve engagement in remote monitoring programs, although its effect on clinical outcomes remains uncertain. However, because our study population was predominantly non-Hispanic White, the broader applicability of these findings to more diverse populations remains uncertain, underscoring the need for future studies in more heterogeneous patient groups.

## Abbreviations

ED: emergency department
MPOTA: MiChart Patient Outreach Texting Application
PEF: patient enrolled flowsheet
